# Advances in Biomarkers for Detecting Early Cancer Treatment-Related Cardiac Dysfunction

**DOI:** 10.3389/fcvm.2021.753313

**Published:** 2021-11-10

**Authors:** Huiyu Xiao, Xiaojie Wang, Shuang Li, Ying Liu, Yijie Cui, Xiaoqin Deng

**Affiliations:** ^1^Department of Radiation Oncology, The First Affiliated Hospital of Dalian Medical University, Dalian, China; ^2^Heart Failure and Structural Cardiology Ward, The First Affiliated Hospital of Dalian Medical University, Dalian, China

**Keywords:** cancer treatment-related cardiac dysfunction, biomarkers, cardio-oncology, anthracyclines, HER2-targeted therapy, immune checkpoint inhibitors, radiotherapy

## Abstract

With the gradual prolongation of the overall survival of cancer patients, the cardiovascular toxicity associated with oncology drug therapy and radiotherapy has attracted increasing attention. At present, the main methods to identify early cancer treatment-related cardiac dysfunction (CTRCD) include imaging examination and blood biomarkers. In this review, we will summarize the research progress of subclinical CTRCD-related blood biomarkers in detail. At present, common tumor therapies that cause CTRCD include: (1) Chemotherapy—The CTRCD induced by chemotherapy drugs represented by anthracycline showed a dose-dependent characteristic and most of the myocardial damage is irreversible. (2) Targeted therapy—Cardiovascular injury caused by molecular-targeted therapy drugs such as trastuzumab can be partially or completely alleviated via timely intervention. (3) Immunotherapy—Patients developed severe left ventricular dysfunction who received immune checkpoint inhibitors have been reported. (4) Radiotherapy—CTRCD induced by radiotherapy has been shown to be significantly associated with cardiac radiation dose and radiation volume. Numerous reports have shown that elevated troponin and B-type natriuretic peptide after cancer treatment are significantly associated with heart failure and asymptomatic left ventricular dysfunction. In recent years, a few emerging subclinical CTRCD potential biomarkers have attracted attention. C-reactive protein and ST2 have been shown to be associated with CTRCD after chemotherapy and radiation. Galectin-3, myeloperoxidas, placental growth factor, growth differentiation factor 15 and microRNAs have potential value in predicting CTRCD. In this review, we will summarize CTRCD caused by various tumor therapies from the perspective of cardio-oncology, and focus on the latest research progress of subclinical CTRCD biomarkers.

## Introduction

Improved early detection methods and the introduction of innovative cancer treatments have allowed a larger number of cancer patients to live longer. There were more than 16.9 million cancer survivors in the United States as of January 1, 2019, a figure that is projected to increase by 30% over the next decade to exceed 22.1 million (1). Long-term adverse events of cancer treatment can affect longevity, and cancer treatment-related cardiovascular injuries are of particular interest. Early-stage breast cancer patients with underlying cardiovascular disease or who have survived for more than 5 years have a higher possibility of dying from cardiovascular disease than of dying from breast cancer (2). Common tumor treatment-related cardiac injuries include left ventricular dysfunction, heart failure (HF), angina, arrhythmia, acute coronary syndromes, thromboembolic ischemia, pericardial disease and myocardial fibrosis (3). Of these, the most serious cardiovascular injuries are cancer treatment-related cardiac dysfunction (CTRCD) and subsequent HF. Chemotherapy, molecular targeted therapy, immunotherapy and radiotherapy can all cause CTRCD. Chemotherapeutic agents such as anthracycline induce CTRCD with dose-dependent ultrastructural changes and irreversible damage, leading to severe HF and death. However, CTRCD induced by molecular targeted therapy drugs (represented by trastuzumab) is independent of drug dose, is not associated with any ultrastructural abnormalities, and can be partially or completely reversed with timely intervention (4). Regardless of whether the damage is reversible or not, the early recognition and prompt treatment of CTRCD are critical to protecting the cardiac function of cancer survivors. It is important to emphasize that not all types of cancer treatment-related cardiotoxicity contribute to heart failure. Fluoropyrimidines and vascular endothelial growth factor receptor targeting drugs often cause coronary spasm, resulting in clinical symptoms such as chest pain and angina on exertion or rest (5).

Experts from the American Society of Echocardiography and the European Association of Cardiovascular Imaging define CTRCD as a decrease in left-ventricular ejection fraction (LVEF) of more than 10%, confirmed by repeated cardiac images at 2–3-week intervals, to a value <53% (6). It should be noted that the diagnostic criteria for CTRCD caused by different solid tumors is the same. However, due to differences between the cancer treatment schemes of different solid tumors, and differences between treatment schemes for the same solid tumors at different stages, the characteristics, prevention and follow-up strategies for CTRCD are different. Echocardiography or radionuclide angiography are typically used to measure LVEF changes in order to diagnose CTRCD. However, the variability of echocardiography measurements, the high false negative rate of radionuclide angiography and the radiation involved in measurements cannot be ignored (7). Ejection fraction-preserved HF is very prevalent in cases of HF. Many other cases do not demonstrate a decline in resting LVEF in the early stages of CTRCD due to myocardial compensation (7). Under these conditions, changes in left-ventricular global longitudinal strain (GLS) appear earlier than CTRD, and GLS is a better predictor of CTRCD than LVEF (8). Over the past two decades, many studies have been performed on CTRCD-related serum biomarkers such as troponin (Tn) and B-type natriuretic peptide (BNP). These serum biomarkers have obvious value in the early identification, assessment and monitoring of CTRCD. In this review we will cover recent literature describing subclinical CTRCD biomarkers.

## Cancer Therapy and CTRCD

### Chemotherapy

Anthracyclines such as doxorubicin, epirubicin and daunorubicin are widely used as chemotherapy for breast cancer, leukemia, lymphoma and stomach cancer. Numerous studies have shown that anthracyclines can cause CTRCD. In a cohort of 2,625 patients treated with anthracyclines followed for 5.2 years, the overall incidence of CTRCD was approximately 9% and most cases occurred within 1 year of treatment (9). The occurrence of CTRCD is related to the cumulative dose of anthracycline (9). Studies have shown that the proportion of patients with adriamycin-related congestive HF is as high as 26% with a cumulative dose of 550 mg/m^2^, and rises to 48% at 700 mg/m^2^ (10). The mechanism through which anthracyclines induce CTRCD is closely related to oxidative stress, the anthracycline-Topoisomerase-II-DNA complex, microRNAs (miRNAs) expression and mitochondrial dysfunction (11–13).

Alkylating agents such as cyclophosphamide, ifosphamide and melphalan form the basis of chemotherapy for solid tumors, leukemia and lymphoma. In a retrospective study of autologous bone marrow transplantation with ifosfamide as part of a combination chemotherapy regimen, 17% of patients developed congestive HF (14). The cardiotoxicity of cyclophosphamide is closely related to the cumulative dose and dose based on body surface area. A dose of 180–200 mg/kg, or 1.5 g/m^2^/d or higher, of cyclophosphamide is a risk factor for CTRCD (15).

The incidence of CTRCD due to taxane therapy was reported to be only 0.7%, which is significantly lower than other chemotherapy drugs (16). Taxanes affect the metabolism of anthracyclines *in vivo*, so when combined with high-dose anthracyclines, the incidence of CTRCD increases to 20% (16). However, the cardiotoxicity of taxane monotherapy was not found to be related to cumulative dose.

### Targeted Therapy

Human epidermal growth factor receptor 2 (Her-2) is a transmembrane glycoprotein encoded by the proto-oncogene *ErBb2* that participates in the proliferation and differentiation of normal tissue cells (17). Her-2 is overexpressed in 15–25% of breast cancer patients, and its overexpression is associated with aggressive growth and a poor prognosis (18). Trastuzumab, a humanized monoclonal antibody against Her-2, is currently the most commonly used targeted drug for metastatic breast cancer and gastric cancer. Anti-Her-2-targeting drugs include pertuzumab, lapatinib and trastuzumab-DM1. Trastuzumab significantly improved the disease-free and overall survival of patients with positive Her-2 breast cancer (18). However, there is a significant risk of cardiotoxicity from trastuzumab-targeted therapy. Early-stage breast cancer patients treated with trastuzumab for 2 years saw a LVEF reduction of 7.2%, while the LVEF of patients treated without only decreased by 0.8% (19). Compared with chemotherapy alone, trastuzumab-combined treatment increased the risk of LVEF decline and congestive HF in patients with early-stage breast cancer 2.17- and 3.71-fold, respectively (20). Trastuzumab-related cardiac dysfunction mainly occurs during medication use, and the cardiac function of most patients returns to normal after standard medical care (19). Trastuzumab-induced CTRCD is mainly due to its targeted inhibition of the neuregulin-1/ErbB pathway, which leads to myofibrillar injury and cardiac systolic dysfunction (21).

### Immunotherapy

Immune checkpoint inhibitors (ICIs) are a primary focus of research on tumor immunotherapy. A total of seven ICIs have been approved for use by the FDA since 2011, including one cytotoxic T lymphocyte-associated protein 4 inhibitor (ipilimumab), three kinds of programmed cell death protein-1 (PD-1) inhibitors (pembrolizumab, nivolumab and cemiplimab), and three kinds of programmed death ligand-1 (PD-L1) inhibitors (atezolizumab, avelumab and durvalumab) (22). These drugs have been particularly beneficial in the treatment of melanoma, Hodgkin's lymphoma, non-small-cell lung cancer and liver cancer. However, the wide range of immune-related adverse events related to ICI use is a challenge. Immunotherapy-related cardiac events are relatively rare, but are often rapidly progressive and potentially fatal, which means that they require close monitoring. Up to now, immune myocarditis has been reported relatively more frequently. Most immunotherapy-induced left ventricular dysfunction is secondary to immune myocarditis. Cases of functional left ventricular dysfunction with no active inflammation, such as dilated cardiomyopathy with left ventricular damage and Takotsubo syndrome with acute left HF, have also been reported (23). In a multicenter study involving 964 patients receiving either one or two ICI treatments, the incidence of immune myocarditis was 1.14% (24) and 49% of patients with immunological myocarditis had LVEF <50% (24). A meta-analysis of 22 clinical trials using single ICIs in the treatment non-small cell lung cancer reported a 2% incidence rate of immune-related HF (25). The symptoms of some patients with immune-associated myocarditis or HF were reversed after timely high-dose corticosteroid treatment, and in some cases LVEF improved to baseline (23). Early recognition and timely treatment are therefore key to the effective management and treatment of immunologic-related cardiac events.

The mechanism behind CTRCD caused by ICIs is currently unclear. ICIs disrupt tumor immune escape and induce activated T cells to attack tumor cells. The same antigenic epitopes are present on both tumor cells and cardiomyocytes, suggesting that ICI activation of T cells against tumor cells also enhances the cross-reaction with cardiac antigens (23).

### Radiotherapy

Radiation-induced heart disease (RIHD) is one of the most serious long-term complications of radiotherapy for thoracic tumors. The emergence of RIHD has led to a reexamination of the benefits and potential risks of radiation therapy. The incidence of HF after contemporary conformal radiotherapy is positively correlated with the mean radiation dose to the heart (26). The likelihood of HF approaches 18% over 20 years when the mean radiation dose to the heart exceeds 3.7 Gy, and is half that in patients who receive less or no radiotherapy (27). Patients are likely to benefit from emerging precision radiotherapy techniques such as intensity-modulated radiotherapy, image-guided radiotherapy and proton therapy, which are able to prevent high radiation doses to the heart. The average time from the end of radiotherapy to the onset of HF was 5.8 years, and more than half of HF cases were ejection fraction-preserved (26). It is therefore not reliable to predict radiation-related cardiac dysfunction based solely on LVEF changes. When radiotherapy is combined with anthracyclines or ICIs, the long-term incidence of cardiac dysfunction is significantly higher than with either treatment alone (28). Clinicians must be aware of the potential cardiotoxicity of combination therapy. Radiotherapy-induced CTRCD is related to endothelial cell injury and reactive oxygen species accumulation, which leads to oxidative stress and mitochondrial dysfunction (29). Myocardial tissue fibrosis is a common pathological outcome of RIHD, and can seriously interfere with left ventricular diastolic function and ejection fraction (30).

## Classical Biomarkers

### Troponin

**Figure 1:**
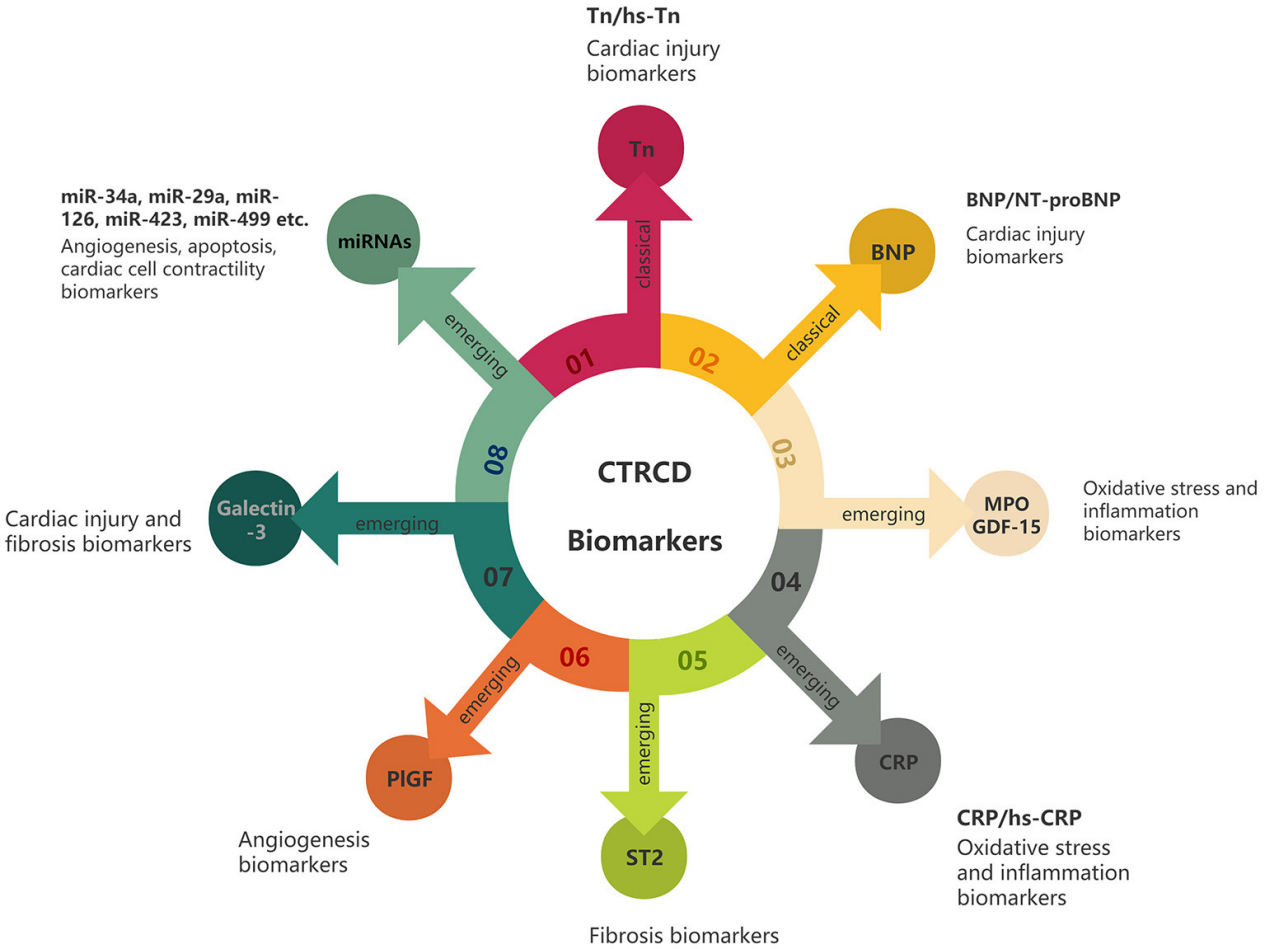
CTRCD biomarkers. Classic biomarkers include Tn and BNP, which mainly reflect cardiac injury. Emerging biomarkers include MPO, GDF-15, CRP, ST2, PlGF, galectin-3 and miRNAs. They can reflect various aspects of the pathophysiology of the heart after cancer treatment, such as oxidative stress, inflammation and fibrosis.

The troponin complex is key to the regulation of skeletal muscle and cardiac muscle thin filament contraction. Cardiac troponin T (cTnT) and cardiac troponin I (cTnI) are expressed exclusively in the myocardium. cTn is released after cardiomyocyte necrosis, and is highly sensitive and specific for diagnosing a myocardial infarction. Increased circulating cTn can precede changes in echocardiographic indicators, and can therefore help in the early diagnosis of subclinical CTRCD (31). High-sensitivity cardiac troponin (hs-cTn) measurements can detect Tn concentrations 10- to 100-fold lower than the original detection method, thus further improving the accuracy and efficiency of myocardial injury diagnosis (32). Compared with hs-cTnT, hs-cTnI has superior early diagnosis potential and is not easily affected by circadian rhythm (33) (Figure 1).

Tn is one of the most popular biomarkers for the prediction of CTRCD. The elevation of Tn before the onset of cardiac symptoms in cancer patients is indicative subclinical functional and myocardial damage, and is closely related to all-cause mortality in cancer patients (34). cTn is a sensitive circulating biomarker for predicting left ventricular hypertrophy after anthracycline treatment. In a 3-year follow-up study, cTnI was significantly elevated during chemotherapy with anthracyclines (35). It reached its peak one month after chemotherapy (26 ng/L), gradually decreased to baseline (3 ng/L) over the proceeding 12 months, then remained at a low level (35). Recent studies have shown that the presence of hs-cTnT levels over 14 ng/L after anthracycline treatment for breast cancer represents a 2-fold increased risk of future CTRCD (36). In contrast, patients with a hs-cTnT level below 5 ng/L at the end of anthracycline treatment did not have CTRCD after 1-year (36).

Multiple studies have shown that Tn levels do not significantly change during trastuzumab monotherapy (36–38). Of 533 invasive breast cancer patients with positive HER-2 after neoadjuvant chemotherapy, only 31 had increased troponin levels during trastuzumab-targeted therapy (37). Patients with elevated cTnI and cTnT levels before trastuzumab had a 2.4- to 4.5-fold increased risk of LVEF decline, although subsequent trastuzumab therapy did not impact this risk (37). The elevated baseline cTnI and cTnT levels were due to cardiac toxicity caused by anthracyclines received prior to the study. Another study of 42 breast cancer patients treated with trastuzumab failed to predict CTRCD using TnT, C-reactive protein (CRP) and BNP measurements (38). However, echocardiography with TVI and strain rate assessment was able to effectively detect preclinical changes in left ventricular systolic function (38). The poor ability of Tn to predict CTRCD efficacy during trastuzumab therapy may be because trastuzumab cardiotoxicity is weaker than that of anthracyclines, and trastuzumab-induced cardiotoxicity is primarily associated with myocardial infarction instead of myocardial injury (21). Interestingly, TnI helped to predict the reversibility of trastuzumab-induced cardiotoxicity. During trastuzumab treatment, all patients with CTRCD whose TnI level was less than 0.08 ng/mL recovered their normal cardiac function, while only 35% of CTRCD patients whose TnI level exceeded this threshold recovered (39).

Tn is the cornerstone of cardiotoxicity screening during ICI therapy. Patients with acute HF during ICI treatment had increased hs-TnT levels and a severely reduced LVEF (40). However, a cardiac MRI did not identify any notable features in these patients they were eventually diagnosed with lymphocytic myocarditis via endocardial biopsy (40). In a study of 35 patients with ICI-related myocarditis, 94% had increased TnT when clinical symptoms developed, and 51% had no significant LVEF abnormalities (41). An imaging examination should therefore not be used as the sole evaluation criteria for ICI-related cardiac events, and Tn is a potential biomarker for predicting ICI-related cardiac toxicity. The study also showed that when TnT was ≥1.5 ng/mL, the risk of cardiovascular death, cardiogenic shock and other major adverse cardiac events increased 4-fold (41). Elevated Tn represents a high-risk of a poor prognosis in patients with ICI-induced cardiotoxicity, although it is not a specific indicator of it. Increased Tn levels during ICI treatment should therefore be interpreted as a warning of an adverse cardiac event, and an immediate oncology–cardiological evaluation should be performed to guide further immunosuppressive therapy (42).

Changes in Tn in patients receiving thoracic radiotherapy has been of recent interest. In a cohort study of 87 patients treated with radiotherapy for breast cancer, lung cancer or mediastinal lymphoma, there was a downward trend in hs-cTnT from pre-radiotherapy to the completion of radiotherapy, especially in the subgroup that received an anthracycline and trastuzumab. This trend was thought to be related to cardiotoxicity caused by the previous treatment (43). However, approximately 15% of patients had an hs-TnT increase of over 30% during radiotherapy, and further studies are needed to explore the relationship between such an increase and the risk of RIHD. Of 58 patients with early left breast cancer who received radiotherapy, 21% had increased hs-cTnT (44). Increased hs-cTnT was positively correlated with the whole heart and left ventricular radiation dose (44). It should be noted that none of these patients received chemotherapy, which rules out the possibility of undetected chemotherapy-induced cardiotoxicity and supports the hypothesis that changes in hs-TnT only represent radiation-induced cardiac injury. In contrast, D'Errico et al. measured TnI before and 5–22 months after radiotherapy for left breast cancer, and it did not exceed the defined positive threshold (45). Tn is inconsistently predictive of radiation-induced cardiotoxicity, so it may not be the best biomarker for diagnosing an acute cardiac injury after radiotherapy. In addition, due to the late onset of most RIHD, the effects of early fluctuations in Tn on the long-term development of heart disease are still yet to be determined by long-term follow-up studies.

Based on existing studies, cTn can identify early myocardial damage caused by chemotherapy or ICIs and predict CTRCD. However, Tn is inconsistently predictive of radiation-induced cardiotoxicity.

### B-Type Natriuretic Peptide

BNP, a neuroendocrine hormone secreted by ventricular myocytes, responsively increases when the ventricular wall stretches due to the increased volume or load stress caused by HF, resulting in beneficial effects such as sodium regulation, diuresis and vasodilation (33). N-terminal proBNP (NT-proBNP), an amino-terminal fragment of BNP, is released into the circulation at an equal proportion to BNP, but has no biologic activity. Compared with BNP, NT-proBNP is stable *in vivo* and *in vitro* and is more popular in clinical practice. Negative BNP and NT-proBNP are used as exclusion criteria for acute and chronic HF, and both are widely used in the diagnosis and prognostic assessment of HF.

There have been numerous reports on the detection of subclinical CTRCD by BNP and NT-proBNP. NT-proBNP is closely related to long-term mortality in select cancer populations, and its serum level gradually increases with tumor progression (34). In a study of 71 breast cancer patients receiving non-high-dose anthracycline chemotherapy, serum NT-proBNP level measurements and echocardiography were performed before each cycle, within 24 h of the completion of chemotherapy, and at 3-, 6- and 12-month follow-ups. A sustained high level of NT-proBNP is associated with future left ventricular dysfunction, while no decrease in LVEF at 1-year follow-up was observed in patients with normal or transiently elevated NT-proBNP levels (46). NT-proBNP reflects reduced cardiac contractile reserve, which is a useful biomarker for detecting subclinical CTRCD. In addition, a cohort study of children with acute lymphoblastic leukemia who received doxorubicin chemotherapy reported that NT-proBNP (an indicator of increased left ventricular wall pressure) may be a more sensitive marker for the detection of subclinical CTRCD than cTnT (an indicator of cardiomyocyte death) (47). However, some researchers believe that the evidence that supports the use of BNP in the prediction of chemotherapy-induced cardiac dysfunction is insufficient, and further clinical studies are required (48).

NT-proBNP successfully predicted the risk of CTRCD in a prospective cohort study that included 323 breast cancer patients who received anthracycline or trastuzumab, with a maximum 3.7-year follow-up (36). Baseline levels of NT-proBNP are strongly associated with CTRCD development, with a 56% increased risk of CTRCD with each doubling of NT-proBNP over baseline values. However, due to the low incidence of CTRCD events in the trastuzumab group, no stratified analysis based on different treatment regimens was performed. Overall, routine screening NT-proBNP measurements are essential to monitoring for CTRCD, especially in those patients who received an anthracycline combined with trastuzumab. However, BNP has performed poorly in some studies. The incidence of cardiac dysfunction was very low in early-stage breast cancer patients who received no anthracyclines and anti-Her-2 therapy alone. In that treatment group the elevation of NT-proBNP was almost undetected (49). The ability of NT-proBNP to predict CTRCD following treatment with anti-Her-2 therapy alone was inferior to its predictive ability following previous exposure to anthracyclines. Some works have found that NT-proBNP was not associated with a reduced LVEF after trastuzumab treatment (38, 50, 51). Despite this, NT-proBNP is an important biomarker for the prediction of CTRCD and its negative predictive value may also be meaningful.

There are few studies that study the use of BNP or NT-proBNP in the prediction of ICI-related cardiac dysfunction. In a study of 30 patients with ICI-related cardiotoxicity, serum BNP levels were elevated in all 14 patients they were measured in (52). In another multicenter study of 35 patients with ICIs-associated myocarditis, 66% of patients had an elevated BNP, but the study did not further analyze the role of an elevated BNP in the development or clinical course of myocarditis (41). While BNP is a potential biomarker for predicting ICI-related cardiac dysfunction, further studies are required.

Elevated BNP levels after radiotherapy have been reported in multiple works, suggesting that it may be a potential biomarker for predicting RIHD. In one study, 25 patients with thoracic cancer received a mean cardiac radiation dose of ≥20 Gy, and elevated BNP levels were detected at the end of radiation therapy and at their first follow-up (1 to 2 months post-radiation) (53). Due to the high cardiac radiation dose used in the study, early changes in BNP may be related to cardiomyocyte inflammation, and radiation may have induced residual diastolic dysfunction. Significant increases in plasma BNP mostly occur at least 9 months after thoracic radiotherapy primarily in patients with radiation-induced myocardial damage (54). An elevated NT-proBNP level was better correlated with ventricular V3Gy, D1cm3/Dmean, and D0.5cm3/D50% than mean heart dose. This suggests that changes in NT-proBNP may be associated with damage caused by a local high dose of ventricular radiation. However, there was almost no difference in NT-proBNP levels before and 20 days after radiotherapy in 87 thoracic cancer patients (43). NT-proBNP therefore may not be effective at predicting acute RIHD. However, as most RIHD occurs late, long-term follow-up may be more useful for evaluating BNP levels after radiotherapy and their predictive value for RIHD.

Based on existing studies, BNP/NT-proBNP can effectively predict chemotherapy-induced cardiac dysfunction, especially delayed HF. BNP/NT-proBNP also has potential in the prediction of targeted therapy or radiation-related cardiac dysfunction.

## Emerging Biomarkers

### MPO

Myeloperoxidase (MPO), an enzyme primarily secreted by neutrophils, plays a vital role in the pathogenesis of atherosclerosis, congestive HF, hypertension and other cardiovascular diseases (55). Elevated MPO usually indicates a high risk of cardiovascular disease and a poor prognosis (55). MPO is an independent predictive factor of 1-year mortality in acute HF patients (56). The over-activation of MPO leads to inflammation and oxidative stress at the cellular level (57). Since oxidative stress plays a key role in the progression of CTRCD, MPO has recently emerged as the most promising biomarker for CTRCD.

Ky et al. first described the utility of MPO in predicting early CTRCD (58). The serum MPO levels of breast cancer patients treated with doxorubicin and paclitaxel followed by trastuzumab were significantly higher than baseline values 3 months after the start of cancer treatment, and gradually decreased over the next 15 months (58, 59). Patients with high MPO levels (422.6 pmol/L) 3 months into cancer treatment had a 36.1% chance of developing CTRCD by 15 months. In addition, MPO combined with TNI was of greater value in the prediction of CTRCD. Specifically, patients with elevated levels of either TnI or MPO had a 31.6 to 33.9% chance of developing CTRCD after 15 months, while patients with elevated levels of both had a 46.5% chance. Demissei et al. showed that high MPO levels prior to the start of anthracycline/trastuzumab therapy were closely associated with an increased risk of CTRCD (36). Each doubling of the baseline MPO level was associated with a 30% increased risk of CTRCD. MPO was more valuable at predicting CTRCD in patients who received a chemotherapy regimen that consisted of doxorubicin combined with trastuzumab compared with monotherapy. Moreover, Todorova et al. found that, of her Her-2-negative breast cancer patients who received doxorubicin chemotherapy and developed subclinical CTRCD had a significantly increased serum MPO level after the first cycle. In contrast, there was no obvious change in the MPO levels of patients with normal LVEF (60). MPO is therefore a potential biomarker of doxorubicin-related cardiotoxicity. The relationship between MPO and ICI-related cardiac dysfunction and RIHD is currently unclear and could be further explored by a cardio-oncologist in the future.

### C-Reactive Protein

C-reactive protein (CRP) is an acute-phase protein that is secreted by the liver during inflammation, and whose expression is regulated by interleukin-6 and tumor necrosis factor-α. A high serum level of CRP (>10 mg/L) can predict the mortality of patients with acute decompensated HF one year after discharge (61). High-sensitivity CRP (hs-CRP) measured using high-sensitivity assays is an independent predictive factor of a poor prognosis for chronic congestive HF patients (62).

CRP/hs-CRP has recently emerged as an inexpensive, readily available and easily repeatable potential biomarker for the identification of early CTRCD. Onitilo et al. monitored cardiotoxicity during trastuzumab treatment in 54 patients with positive Her-2 breast cancer who had received standard chemotherapy (51). When the hs-CRP level was ≥3 mg/L, its sensitivity and specificity was 92.9 and 45.7%, respectively. The negative predictive value hs-CRP for patients with normal levels (hs-CRP <3 mg/L) was 94.1%. This suggests that most patients with normal hs-CRP levels can rule out the risk of future LVEF reduction, while patients with abnormal hs-CRP levels need to be strictly monitored during follow-up. The maximum hs-CRP value was detected at a median of 77.5 days before significant LVEF decline, and most subjects developed cardiotoxicity within 90 days of their peak hs-CRP level. Notably, the study evaluated both TnI and BNP, suggesting that neither were very potent biomarkers in their subjects. Similarly, Todorova et al. observed that the CRP levels of patients with decreased LVEFs after one cycle of doxorubicin chemotherapy were visibly higher than those who had no change in their LVEF (60). Considering that CRP is associated with surgery and infection, the negative predictive value of combined cTnI and CRP biomarkers is higher than either value alone. Other studies also found no clear association between CRP/hs-CRP and CTRCD induced by either chemotherapy or targeted therapy (38, 58).

CRP has recently been shown to have important predictive value in tumor immunotherapy. CRP is a prognostic biomarker for ICI treatment (63, 64). Metastatic renal cell carcinoma patients with high CRP levels (CRP ≥ 2.1 mg/dL) treated with nivolumab had a shorter progression-free survival and overall survival than those with low CRP levels (63). Similarly, elevated CRP levels in metastatic melanoma patients treated with ICIs suggests a poor prognosis (64). CRP can also predict tumor response to ICI treatment (65), and plays an important role in the prediction of immune-related adverse events. Abolhassani et al. analyzed 88 immune-related adverse events in 37 melanoma patients, which included cardiovascular adverse events, cutaneous adverse events and endocrine adverse events, and 93% of which were related to a 6.3-fold increase in CRP (66). An elevated CRP was observed in 42% of patients who had adverse events prior to the development of any clinical symptoms. There are few reports on the use of CRP for predicting ICIS-related cardiac adverse events, but existing studies suggest that CRP is a potential biomarker for these as well.

hs-CRP may also be a biomarker for radiation-induced cardiac dysfunction. Canada et al. found reported increased hs-CRP in 64% of 25 patients 1.8 years after the completion of thoracic radiotherapy (67). Abnormal hsCRP was significantly associated with decreased LVEF and reducede cardiac reserve. However, CRP was not very effective at predicting cardiac events during radiotherapy (68). Lipshultz et al. demonstrated found that hs-CRP level was higher in childhood cancer survivors than in normal controls regardless of exposure to cardiotoxic treatment (chemotherapy or radiotherapy) (69). Moreover, hs-CRP level was shown to be negatively correlated with left ventricular mass, wall thickness and dimension. Systemic inflammation may further impair cardiac function in childhood survivors, so hs-CRP is expected to be an effective biomarker for monitoring the cardiovascular condition of cancer survivors.

CRP/hs-CRP demonstrated good CTRCD screening ability in the setting of chemotherapy, targeted therapy and immunotherapy. Of the emerging CTRCD biomarkers, CRP/hs-CRP has a relatively high predictive value and is expected to be widely used in clinical practice in the future.

### ST2

ST2, a member of the interleukin 1 receptor family, is highly expressed when cardiac muscle cells are subjected to mechanical stress. The soluble receptor form of ST2 (sST2) is present in human circulation, and is an effective indicator of myocardial cell extension and fibrosis. sST2 levels are closely associated with sudden cardiac death and long-term mortality in HF patients (70, 71).

Few studies have addressed the predictive value of ST2 for CTRCD. Sawaya et al. followed 81 Her-2-positive breast cancer patients treated with anthracyclines followed by taxanes and trastuzumab or radiation for 15 months (50). ST2 levels were higher than normal serum reference values at baseline, but did not change significantly throughout the follow-up period (50, 72). Recent studies have suggested that there is a possible association between sST2 and radiation-induced cardiotoxicity. Zeng et al. assessed serum sST2 levels in 60 patients receiving chest radiotherapy (73). sST2 levels increased gradually over the course of radiotherapy, but no significant changes were observed in BNP or LVEF. It is worth noting that the ST2 change rate was positively correlated with heart V5, V10 and V20, and mean heart radiation doses. sST2 may therefore be useful in detecting acute radiological cardiotoxicity. Aula et al. grouped 63 breast cancer patients receiving radiotherapy via the severity of GLS deterioration, and correlated this with changes in sST2 levels (74). Patients with a GLS decline greater than 15% had a small but significant rise in sST2 at baseline, after radiotherapy, and three years after radiotherapy. Patients with a reduction in GLS of less than 15% had no significant fluctuation in sST2 level. sST2 may therefore be a potential biomarker for predicting radiation-related cardiotoxicity.

### GDF-15

Growth differential factor 15 (GDF-15) is a member of the transforming growth factor beta superfamily that has cell protection, anti-apoptosis and anti-hypertensive properties. GDF-15 is upregulated in the setting of inflammation, oxidative stress, tissue hypoxia and cell injury (75). High serum GDF-15 levels in HF patients suggest a poor prognosis, and are predictive of 1- to 5-year mortality (76). Serum concentrations of GDF-15 were found to increase continuously during the 15-month follow-up of breast cancer patients treated with doxorubicin and trastuzumab, and increased GDF-15 levels were significantly associated with an increased risk of LVEF reduction (59). The GDF-15 levels of patients with mediastinal lymphoma or lung cancer who received thoracic radiotherapy were significantly higher after radiotherapy than before radiotherapy (1171ng/L increased to 1887ng/L) (43). However, early changes in GDF-15 were not observed in breast cancer patients, which may be related to the lower dose of radiation to the heart that is received during breast cancer radiotherapy. Moreover, changes in GDF-15 in this study were not associated with changes in LVEF during radiotherapy. Long-term follow-up studies may help define the predictive value of elevations in GDF-15 in the setting of radiation-related cardiotoxicity. It is worth noting that GDF-15 has been found to be upregulated during tumorigenesis and progression, so the value of GDF-15 in the prediction of CTRCD must be carefully considered (77).

### PlGF

Placental growth factor (PlGF) is a member of the vascular endothelial growth factor family. PlGF promotes the angiogenesis of ischemic heart and limb tissue by specifically binding with the Flt-1 receptor, and also participates in the pathologic processes of atherosclerosis and arthroinflammation (78, 79). It is worth noting that antagonistic PIGF/Flt-1 signaling can effectively inhibit tumor angiogenesis (79). PlGF is elevated in coronary heart disease and HF and is thought to have promising prognostic properties. High PlGF levels on admission can predict the long-term poor prognosis of acute decompensated HF (80). Putt et al. found that PlGF was consistently elevated and predictive of CTRCD in patients who received an anthracycline and trastuzumab (59). The potential predictive ability of PlGF may be related to the inhibitory effects of trastuzumab on angiogenesis (81). In addition, PlGF was acutely elevated in patients who received chest radiotherapy, and PIGF level was correlated with cardiac radiation dose (43). PlGF may therefore be a potential biomarker for predicting RIHD.

### Galectin-3

Galectin-3, a member of the beta-galactoside-binding lectin family, is involved in the occurrence and development of cardiac fibrosis, HF and atherosclerosis (82). Galectin-3, secreted by macrophages, is key to heart remodeling (82). Numerous studies have shown that galectin-3 levels predict the risk of death in HF patients (83). Galectin-3 is upregulated in cancer cells, and promotes cancer progression and metastasis (84). Galectin-3 inhibitors can effectively block lung adenocarcinoma growth and metastasis, and increase the efficacy of PD-L1 ICIs (85). In animal studies, cardiac dysfunction induced by doxorubicin was accompanied by a massive accumulation of galectin-3, and cardiac function improved when galectin-3 was inhibited (86). Galectin-3 is expected to become a new therapeutic target for cancer and CTRCD. The expression of galectin-3 was upregulated in macrophages *in vitro* 24 h after radiation exposure (87). In contrast, Ky et al. monitored the galectin-3 serum concentrations in breast cancer patients during and after treatment with doxorubicin and trastuzumab and found no significant changes in galectin-3 levels throughout treatment (58). Moreover, galectin-3 could not effectively predict LVEF decline in their study. Altena et al. performed echocardiography and serum galectin-3 measurements in testicular cancer survivors who received cisplatin a median of 10 months and 6.9 years after chemotherapy (88). Cardiac abnormalities during long-term follow-up were more likely to manifest as diastolic dysfunction than systolic dysfunction. Galectin-3 levels were markedly lower 6.9 years after chemotherapy than at baseline and 10 months, suggesting that the decreased diastolic function was not associated with cardiac fibrosis. The unsatisfactory predictive value of galectin-3 for CTRCD in humans may be related to the inability of echocardiography to accurately assess myocardial fibrosis. Cardiac magnetic resonance imaging may be a better imaging tool for assessing this outcome.

### MicroRNAs

MicroRNAs (miRNAs) are highly conserved, single-stranded non-coding RNAs approximately 22 nucleotides in length that bind to messenger RNAs to silence gene expression. miRNAs participate in the regulation of approximately 30% of essential human gene expression, meaning that miRNAs regulate a large number of cell metabolic processes such as proliferation, apoptosis and metastasis (89). The combination of miRNAs and NT-probNP can accurately identify non-acute HF with retained ejection fraction (90). miRNAs are tissue-specific, relatively stable, easily stored at room temperature and can be accurately measured using quantitative real-time PCR and other techniques. miRNAs are therefore a promising biomarker for CTRCD.

miRNAs have recently been used to detect subclinical chemotherapy-related cardiotoxicity. Leger et al. observed a sharp increase in plasma miR-29b and miR-499 levels within 6 to 24 h of chemotherapy in children with an anthracycline chemotherapy-associated acute myocardial injury, and a significant correlation between elevated miRNAs and anthracycline dose (91). Lakhani et al. indicated that circulating levels of miR-34a, miR-29a, miR-126, miR-423 and miR-499 were upregulated in breast cancer patient 3 or 6 months after anthracycline treatment (31). There was a significant correlation between the upregulation of miRNAs and increased serum hs-cTn, a sign of myocardial injury. miRNAs can be used to effectively detect subclinical cardiac dysfunction caused by anthracyclines before LVEF decreased (31). Changes in miRNAs may be related to their involvement in regulating cardiac function. Not only does miR-34a activate the miR-34a-5p/SIRT1/p66SHC pathway to enhance cardiomyocyte apoptosis, it also increases the release rate of pro-inflammatory factors such as TNF-a and IL-6, thus playing an important role in the progression of anthracycline-induced cardiotoxicity (92). miR-29 is upregulated to inhibit the myocardial fibrosis response, which is an important aspect of cardiac remodeling after myocardial injury (93). miR-126 is expressed in endothelial cells and accelerates angiogenesis, and its upregulation after chemotherapy may be due to cellular stress and the anti-angiogenesis effects of chemotherapy drugs (94). The overexpression of miR-423 after chemotherapy directly targets O-GlcNAc transferase and induces apoptosis in cardiomyocytes, thus leading to HF (95). miRNAs are therefore promising new tools for detecting early chemotherapy-related cardiac dysfunction.

Recent studies suggest that increased levels of miR-130a during anthracycline plus trastuzumab treatment are an independent predictor of cancer treatment-related cardiotoxicity (96). Notably, Horie et al. proposed that miR-146a was significantly upregulated to inhibit ERBB4 expression after the application of doxorubicin. Although this inhibition is temporary, it blocks the activity of the NRG-1/ErbB signaling pathway and aggravates cardiomyocyte death. Combined treatment with trastuzumab, an ERBB2 inhibitor, aggravates the cardiotoxicity of the cancer treatment and further triggers HF (97). However, recent studies have shown that the administration of miR-146a-rich exosomes can not only alleviate doxorubicin/trastuzumab-induced oxidative stress in cardiomyocytes, but can also enhance the silencing effects of miR-146a on some target genes that encode the signaling mediators of the inflammation and cell death axis (98). miR-146a may therefore play a protective role in doxorubicin/trastuzumab-induced cardiotoxicity (98).

miRNAs have been found to be upregulated in animal models of autoimmune myocarditis, and correlated with the development of autoimmune myocarditis (99). miRNAs also play an important role in ICI-induced cardiac injury. Specifically, the PD-1 inhibitor promote a large amount of miR-34a aggregation in cardiomyocytes by regulating the miR-34a/KLF4 and miR-34a/Pnuts signaling pathways to induce myocardial inflammation and promote myocardial aging (100, 101). miRNAs are therefore potential therapeutic targets for ICI-induced cardiac injury.

Numerous studies have noted a close relation between miRNAs and RIHD. Hawkins et al. measured the circulating miRNA levels of 63 patients with NSCLC before radiotherapy and found that elevated miRNAs, such as miR-574, were correlated with a greater risk of radiation-related cardiotoxicity (102). In addition, the expression of miR-29a and miR-150 decreased with an increased radiation dose during chest radiotherapy (103). Decreased miR-29a during a myocardial infarction can enhance the cardiac fibrosis response (104). The changes in miR-29a during chest radiotherapy may be related to the pre-fibrotic state after radiation, making miR-29a is a potential biomarker for predicting radiation-related cardiotoxicity. A large number of animal studies have found that miR-21 is the most significantly upregulated miRNA in irradiated cardiomyocytes, and that its expression nearly doubles after radiation (105). The expression of miR-21 can inhibit apoptosis and promote cell proliferation, which is beneficial to the early resistance of cardiomyocytes to radiation injury. It is important to note that the expression of miR-1 in myocardial cells was significantly decreased after radiation, especially in the left ventricle (105). miR-34a was upregulated in irradiated cardiomyocytes and promoted cell senescence through the miR-34a/sirtuin 1 signaling pathway (106). miRNAs are important cardiovascular regulatory factors, and miRNA profiling before, during and after radiotherapy may provide more predictive and prognostic information about radiation-related cardiotoxicity.

In sum, miRNAs are a new tool for detecting early CTRCD. miRNAs profiles can provide a great deal of information about CTRCD, especially in those who have received chemotherapy and radiotherapy.

### Other Emerging Biomarkers

There are many other clinical biomarkers that may be useful in the prediction of CTRCD. Glycogen phosphorylase BB (GPBB), one of the subtypes of glycogen phosphorylase, is mainly distributed in the heart and brain. GPBB is the key enzyme in glycogenolysis, supplying energy to cardiomyocytes during myocardial ischemia (107). GPBB is released into the plasma via the cell membrane, which has increased permeability during myocardial ischemia. Horacek et al. observed that GPBB significantly elevated six months after high-dose anthracycline chemotherapy, and was associated with left ventricular diastolic dysfunction (108). In contrast, the levels of other biomarkers (such as myoglobin and heart-type fatty acid-binding protein) did not significantly fluctuate. GPBB may therefore be an emerging biomarker for predicting CTRCD whose potential requires further confirmation in large prospective studies.

Anthracycline-induced CTRCD is closely associated with oxidative stress and endothelial dysfunction. The arginine–nitric oxide metabolic pathway plays an important role in these two pathophysiological processes. Arginine–nitric oxide metabolites such as asymmetric dimethylarginine and N-monomethylarginine can inhibit the activity of endothelial nitric oxide synthase and cause DNA damage, myocardial cell apoptosis and endothelial cell dysfunction (109). In a follow-up study of 170 breast cancer patients treated with doxorubicin and/or trastuzumab, the serum concentrations of dimethylargeinine and N-monomethylargeinine were significantly increased soon after chemotherapy (110). Changes in the serum levels of arginine–nitric oxide metabolites were correlated with an increased risk of CTRCD over 5.4 years of follow-up. As inflammation and immune response are essential to the early stages of doxorubicin-induced cardiotoxicity, the high expression of immune response proteins such as chemokines and matrix metalloproteinases may be related to the sensitivity of individual cardiomyocytes to doxorubicin (111). Doxorubicin-induced CTRCD is more likely to occur in patients with abnormal immune response proteins, so immune response proteins could be predictive biomarkers of CTRCD. Moreover, as carriers of some proteins and genetic materials, exosomes can aid in the early diagnosis of cardiotoxicity induced by doxorubicin (112). It is noteworthy that the serum lipopolysaccharide-binding protein could be used to predict diastolic dysfunction 3 years after radiotherapy, and is a promising predictor of radiation-related cardiac dysfunction (113) (Table 1).

**Table 1:** Researches on emerging biomarkers for CTRCD.

**Reference**	**Biomarkers**	**Treatment**	**Patient population**	**Positive results**
Lakhani et al. 2021 (31)	MiR-34a, MiR-29a, MiR-126, MiR-423, MiR-499	Doxorubicin	17 breast cancer patients with triple negative status	Circulating miRNAs were upregulated at 3 or 6 months after treatment and effectively detected CTRCD before LVEF decreased
Demissei et al. 2020 (36)	MPO, Thrombomodulin, thrombin-antithrombin complex,nucleosomes, CRP	Doxorubicin,cyclophosphamide, paclitaxel and/or trastuzumab	323 breast cancer patients	Each doubling of baseline MPO level was associated with a 30% increased risk of CTRCD
Demissei et al. 2019 (43)	PlGF and GDF-15	Radiotherapy	87 breast cancer, lung cancer, or mediastinal lymphoma patients	PlGF and GDF-15 were significantly increased during radiotherapy in lung cancer/lymphoma patients
Onitilo et al. 2012 (51)	hs-CRP	Trastuzumab	54 Her-2+ Breast Cancer patients	Normal hs-CRP levels may be associated with low future risk for decreased LVEF
Ky et al. 2014 (58)	CRP, GDF-15, MPO, PlGF, galectin-3 and soluble fms-like tyrosine kinase receptor(sFlt)-1	Doxorubicin, Cyclophosphamide, Paclitaxel, Trastuzumab	78 Her-2+ Breast Cancer patients	Levels of CRP, GDF-15, MPO, PlGF and sFlT-1 were significantly higher than baseline three months after the start of treatment, and elevated MPO levels were associated with an increased risk of CTRCD
Putt et al. 2015 (59)	MPO, PlGF and GDF-15	Doxorubicin, Cyclophosphamide, Paclitaxel, Trastuzumab	78 Her-2+ Breast Cancer patients	Increased levels of MPO, PlGF and GDF-15 were associated with an increased risk of CTRCD
Todorova et al. 2020 (60)	MPO, Thrombomodulin, thrombin-antithrombin complex,nucleosomes, CRP	Doxorubicin, Cyclophosphamide	51 early Her-2- breast cancer patients	The levels of MPO, CRP and thrombin-antithrombin complex were significantly increased in patients with subclinical CTRCD before and after the first cycle of chemotherapy, while there were no significant changes in patients with normal LVEF
Canada et al. 2020 (67)	hs-CRP	Radiotherapy	25 breast or lung cancer patients	hs-CRP was elevated in 64% of patients 1.8 years after radiotherapy, and the elevated hsCRP level was significantly correlated with decreased LVEF and smaller cardiac reserve.
Zeng et al. 2020 (73)	sST2	Radiotherapy	60 thoracic malignancy cancer patients	Serum sST-2 levels were elevated over time during radiotherapy. Heart V5, V10, V20 and mean heart dose were independently and positively associated with the elevated ST-2 change rate.
Aula et al. 2020 (74)	sST2	Radiotherapy	53 breast cancer patients	Patients with a GLS decline greater than 15% showed a small but significant increase in sST2 at baseline, after radiotherapy, and three years after radiotherapy, while patients with a GLS decline less than 15% showed no significant fluctuation in sST2 levels.
Frères et al. 2018 (94)	sST2, miR-126, miR-199a, miR-423, miR-34a	Cyclophosphamide, epirubicin and paclitaxel±trastuzumab or lapatinib	45 breast cancer patients	The levels of sST2 and miRNAs were significantly increased
Feng et al. 2021 (96)	miR-130a	Epirubicin/cyclophosphamide, docetaxel plus trastuzumab	72 Her-2+ breast cancer patients	Elevated miR-130a was an independent risk predictor for CTRCD
Hawkins et al. 2019 (102)	14 circulating miRNAs species	Radiotherapy	63 non-small cell lung cancer patients	Elevated levels of miRNAs, represented by miR-574, suggest an increased risk of radiation-associated cardiotoxicity
Dinh et al. 2016 (103)	circulating miRNAs	Radiotherapy	5 non-small cell lung cancer patients	The expression of miR-29a and miR-150 decreased with the increase of radiation dose
Horacek et al. 2013 (108)	GPBB, myoglobin and heart-type fatty acid binding protein	Anthracyclines	24 acute leukemia patients	A significant increase in GPBB levels after chemotherapy continued until 6 months after chemotherapy and the increased GPBB was significantly associated with left ventricular diastolic dysfunction
Finkelman et al. 2017 (110)	arginine, citrulline, ornithine, asymmetric dimethylarginine (ADMA), symmetric dimethylarginine (SDMA), and N-monomethylarginine (MMA)	Doxorubicin ± trastuzumab	170 breast cancer patients	Changes in serum levels of arginine-nitric Oxide Metabolites were associated with an increased risk of CTRCD at up to 5.4 years of follow-up
Yu et al. 2018 (111)	40 distinct chemokines, 9 matrix metalloproteinases and 33 potential markers of cardiovascular diseases	Doxorubicin	27 breast cancer patients	Patients with abnormal immune response proteins were more likely to develop CTRCD
Chalubinska-Fendler et al. 2019 (113)	lipopolysaccharide-binding protein, fatty acid binding protein, CRP	Radiotherapy	129 breast cancer patients	Lipopolysaccharide Binding Protein could predict diastolic dysfunction 3 years after radiotherapy

### Biomarkers and Management of CTRCD

Prevention strategies for CTRCD currently include: (1) Correction of pre-existing cardiovascular risk factors; (2) Direct reduction of cardiotoxicity: through a reduced cumulative dose of chemotherapy drugs, use of anthracycline liposomes and altered administration regimens; (3) Cardioprotectants such as dexrazoxane, which has been approved by the FDA for the cardiac protection of metastatic breast cancer patients receiving high doses of doxorubicin. (4) Cardiovascular support drugs such as angiotensin-converting enzyme inhibitor/angiotensin receptor blocker, selected beta blockers and statins have been shown to prevent CTRCD (114). Tn provides important guidance during the development of a CTRCD preventive strategy. Early use of enalapril in patients with elevated Tn after high-dose chemotherapy can effectively prevent cardiotoxicity and improve CTRCD prognosis (115). The prophylactic use of enalapril even after the development of troponin abnormalities can effectively reduce the incidence of left ventricular dysfunction, with no significant difference between the two strategies (116). Beta-blockers can help patients who have already developed trastuzumab-induced cardiac dysfunction continue to complete targeted therapy (117). An angiotensin-receptor-neprilysin inhibitor that significantly improves cardiovascular outcomes was a breakthrough in the treatment of HF, and is expected to be used in the treatment of CTRCD in the future. ESMO consensus recommendations clearly state that asymptomatic patients receiving cardiotoxic anti-cancer therapy should seek the help of cardio-oncologists immediately after the detection of an elevated Tn, and it is recommended that patients be evaluated for LVEF and GLS and cardiac protective therapy started after the exclusion of ischemic heart disease (114). Patients with elevated blood markers without significant cardiac dysfunction can continue anti-cancer therapy under close monitoring. However, clinicians still rely on biomarkers to guide the management of CTRCD rather than hierarchical management. The normal threshold of NT-proBNP for diagnosing heart failure varies between different age groups, and should be of particular interest to clinicians who wish to guide the management of CTRCD.

## Conclusion and Prospect

In this review, we emphasize the vital role of serum biomarkers in the detection of subclinical CTRCD. We summarized the progress of research on two classical biomarkers (Tn and BNP) and several emerging biomarkers (MPO, CRP, ST2, GDF-15, PlGF, Galectin-3, miRNAs and GPBB) in the prediction of early CTRCD. Classical biomarkers have good sensitivity and specificity, low cost, and provide quantifiable and reproducible data. A large number of studies have confirmed that classical markers can predict the risk of CTRCD and guide further treatment. Emerging biomarkers are involved in many biological processes, and their addition may further enhance the utility of biomarkers in the management of CTRCD. For example, CRP and ST2 are associated with inflammation, galectin-3 is associated with cell proliferation, MPO and GDF-15 are associated with oxidative stress, PlGF is associated with angiogenesis and miRNAs are associated with apoptosis. However, disadvantages of emerging biomarkers are their high cost and low popularity (Table 2).

**Table 2:** Predictive value of biomarkers for cancer treatments.

	**Chemotherapy**	**Targeted therapy**	**Immunotherapy**	**Radiotherapy**
Tn/hs-Tn	+++	+	+++	++
BNP/NT-pro BNP	+++	++	+	++
MPO	++	++	-	-
CRP/hs-CRP	++	++	++	+
ST2	-	-	-	++
GDF-15	+	+	-	+
PlGF	+	+	-	+
Galectin-3	+	-	-	-
miRNAs	++	+	+	++
GPBB	+	-	-	-
Arginine-nitric oxide metabolites	+	+	-	-
Immune response proteins	+	-	-	-
Lipopolysaccharide-binding protein	-	-	-	+

*(−) Indicates that there is currently no research report on the relationship between biomarkers and cancer treatments. (+) Indicates that biomarkers have been reported to be associated with cancer treatment. (++) Represents a large number of studies reported an association between biomarkers and cancer treatment. (+++) Represents that biomarkers are recognized as predictors of cancer treatment*.

In addition to blood biomarkers, cardiac imaging has been widely used in the detection of subclinical CTRCD. Echocardiography, currently the most commonly used imaging tool for detecting CTRCD in clinical practice, is non-invasive, highly repeatable, simple and safe. LVEF is the most widely used cardiac function indicator in echocardiography, and can independently predict the short-term and long-term mortality of CTRCD. However, due to the early compensatory response of LVEF after heart injury, subclinical CTRCD cannot be accurately detected by LVEF. Strain-echocardiography is a new tool for evaluating myocardial deformation. GLS, as assessed by speckle-tracking echocardiography, can accurately measure early myocardial changes caused by cancer treatment. A 10–15% reduction in GLS is the most effective predictor of early CTRCD (3). A large number of studies have shown that GLS can effectively detect CTRCD before LVEF changes. The randomized controlled trial by Thavendiranathan et al. showed that compared with LVEF, preventive cardioprotective therapy based on GLS decline can significantly reduce the incidence of CTRCD (118). GLS is increasingly being used by clinicians to monitor the cardiac function of patients undergoing cancer treatment at baseline, during treatment, and during follow-up. However, the detection of GLS in elderly patients, obese patients and patients with valvular heart disease and coronary artery disease is limited (119). Although a few of the studies mentioned above have shown that GLS can predict CTRCD before blood markers change, the competition between GLS and blood markers for predictive ability requires further exploration. A combination of blood markers and imaging techniques may be the best choice for predicting CTRCD. The sensitivity of hs-TnI combined with longitudinal strain measurements was significantly higher than that of simple biomarker measurements (74% increased to 87%), with a negative predictive value of 91% (50). NT-proBNP and GLS measurements also help to improve the detection rate of early CTRCD, and this mode can successfully detect CTRCD before LVEF declines and clinical symptoms appear (120). It is necessary to note that during the COVID-19 pandemic, patients without symptoms associated with CTRCD can be monitored over extended imaging intervals (121). In order to reduce the risk of exposure, cardiac toxicity can be monitored mainly using routine blood markers. CTRCD was further evaluated by imaging when blood markers were abnormal.

Most clinical studies on the prediction of early CTRCD focused on the traditional biomarkers Tn and BNP, with few studies on emerging markers. Future studies can be improved in the following ways: (1) a longer follow-up time will provide valuable data for improve the predictive power of biomarkers for late cardiotoxicity; (2) increasing the sample size will improve study accuracy; (3) the direction of cardiotoxicity caused by targeted therapy or ICIs should be further explored. Genetic testing, human stem cell-derived cardiomyocytes and artificial intelligence have all been used to predict subclinical CTRCD (122).

In conclusion, the early diagnosis, treatment and prognostic assessment of CTRCD in the era of precision medicine all require close cooperation between oncologists and cardiologists. Emerging biomarkers have strong potential to predict subclinical CTRCD and provide direction for future cardio-oncology.

## Author Contributions

All authors contributed to the conception, development, and critical appraisal of this manuscript. All authors contributed to the article and approved the submitted version.

## Funding

This study was funded by Natural Science Foundation of Liaoning Province (No. 2012049) and Youth Foundation of Dalian (Nos. 2017QN006 and 2019QN028).

## Conflict of Interest

The authors declare that the research was conducted in the absence of any commercial or financial relationships that could be construed as a potential conflict of interest.

## Publisher's Note

All claims expressed in this article are solely those of the authors and do not necessarily represent those of their affiliated organizations, or those of the publisher, the editors and the reviewers. Any product that may be evaluated in this article, or claim that may be made by its manufacturer, is not guaranteed or endorsed by the publisher.
